# Percutaneous rotational osteotomy of the femur utilizing an intramedullary rod

**DOI:** 10.1007/s11751-016-0257-3

**Published:** 2016-06-18

**Authors:** Peter M. Stevens, Christian J. Gaffney, Heather Fillerup

**Affiliations:** University of Utah, Salt Lake City, UT USA

**Keywords:** Anteversion, Retroversion, Femoral osteotomy, Intramedullary rod, Osseous necrosis

## Abstract

The purpose is to describe the technique and report the results and complications of percutaneous femoral rotational osteotomy, secured with a trochanteric-entry, locked intramedullary rod, in adolescents with femoral anteversion. Our series comprised an IRB approved, retrospective, consecutive series of 85 osteotomies (57 patients), followed to implant removal. The average age at surgery was 13.3 years (range 8.8–18.3) with a female-to-male ratio of 2.8:1. The minimum follow-up was 2 years. Eighty-three osteotomies healed primarily. Two patients, subsequently found to have vitamin D deficiency, broke screws and developed nonunions; both healed after repeat reaming and rod exchange and vitamin supplementation. Preoperative symptoms, including in-toeing gait, tripping and anterior knee pain or patellar instability, were resolved consistently. We did not observe significant growth disturbance or osteonecrosis. We noted a 12.5 % incidence of broken interlocking screws; this did not affect the correction or outcome except for the two patients mentioned above. This prompted a switch from a standard screw (core diameter = 3 mm) to a threaded bolt (core diameter = 3.7 mm). These results have led this technique to replace the use of plates or blade plates for rotational osteotomies.

## Introduction

Persistent femoral anteversion is a frequent contributing factor to anterior knee pain and patello-femoral instability in adolescents. If left untreated, there may be a susceptibility to acute or repetitive injuries that limit sports participation and lifestyle. Presenting symptoms include anterior knee pain and patellar mal-tracking or frank instability. While retroversion is less common and does not cause knee pain typically, it represents an indication for surgical treatment occasionally. Non-operative measures such as NSAIDs, knee braces and physical therapy are palliative at best. The definitive treatment comprises rotational osteotomy of the femur. Once the osteotomy has healed, unrestricted activities are permitted and the rod is removed at an average of 12 months post-osteotomy.

## Patients and methods

This is a consecutive series of patients who underwent correction of anteversion between 2010 and 2014. All patients were older than 8 years and had presented with persistent in-toeing, tripping and anterior knee pain with or without patello-femoral instability. The symptoms had been refractory to non-operative management. There are several advantages to deferring osteotomy until after the age of 8 years:

1. there is torsional remodeling potential that may mitigate against the need for surgery before the age of 8 years,
2. after the age of 8 years, recurrence of torsion is rare so an osteotomy need not be repeated,
3. femoral osteotomy (or fracture) in younger children may stimulate overgrowth resulting in iatrogenic limb length inequality,
4. there is diminished likelihood of altering trochanteric growth or causing coxa valga,
5. there is appropriate, transtrochanteric instrumentation available that permits stable, antegrade intramedullary nailing of the femur [1, 2].

By employing a quadriceps-sparing, percutaneous osteotomy, the blood loss is minimal and healing rapid with morbidity decreased. The purpose of this review is to report our experience with this less invasive form of osteotomy as compared to the more established or popular plating or blade plate fixation methods, and include complications and outcomes from a single surgeon series.

With IRB approval, a retrospective review was conducted of 57 patients who underwent a percutaneous osteotomy of the femur to correct anteversion (or retroversion) utilizing a trochanteric-entry intramedullary rod for fixation. We evaluated historical complaints and functional limitations as well as clinical findings including anterior knee pain, patellar tracking, gait pattern, comparing the preoperative status to that at the time of rod removal. This cohort comprised 57 patients (15 males and 42 females) ranging in age from 8.8 to 18.3 years (average 13.3 years) at the time of the osteotomy. Bilateral correction was undertaken in 28 and a unilateral osteotomy in the other 29, giving a total of 85 osteotomies. Simultaneous surgical procedures included rotational osteotomy for tibial torsion and guided growth for genu valgum or fixed knee flexion deformity. The etiology of femoral torsion was idiopathic in 53 patients and neurogenic in four. All patients were seen in follow-up until rod removal; this was at an average 10.7 months (range 9–12 months) following osteotomy.

The physical examination included measurement of limb lengths, frontal knee alignment (varus/valgus), observation of the gait pattern and prone torsional profile, noting the degrees of inward and outward hip rotation and the thigh/foot axis. We did not recommend surgery unless the degree of excessive femoral torsion (beyond the normal 11°) measured greater than 20°. The gait pattern was observed with attention to both the foot progression angle and the knee progression angle. The foot progression angle may appear normal in patients with concomitant outward tibial torsion. However, the effects of inward femoral and outward tibial torsion are cumulative, not compensatory, with respect to the patella and knee. For example, a patient with 30° of femoral anteversion and 30° outward thigh–foot axis (tibial torsion) is subject to 60° of torsional stress on the knee. For these patients, simultaneous femoral and tibial osteotomies are justified. We elected not to employ PROMs (patient-reported outcomes) because we did not have an adequate tool to apply for this retrospective series.

**Fig. 1:**
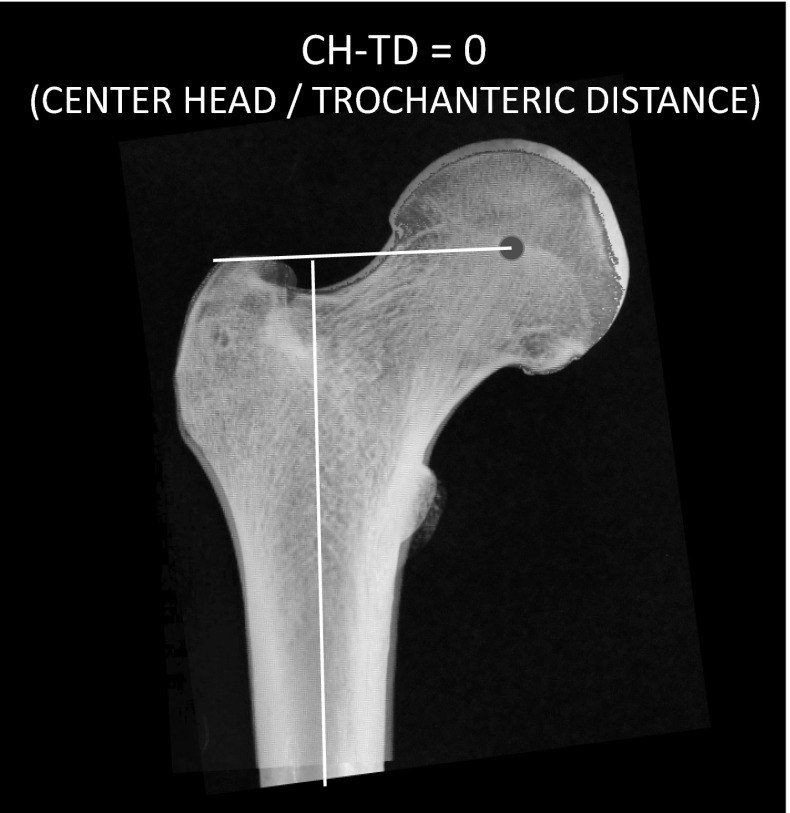
The center of femoral head to trochanteric tip vertical offset is normally zero. This affords maximum efficiency for the abductors. In preadolescent children, this distance is not measurable on plain radiographs because the tip of the trochanter is not ossified. In our series, transtrochanteric rod insertion did not result in any observed change in the center head/trochanteric distance (CH–TD)

**Fig. 2:**
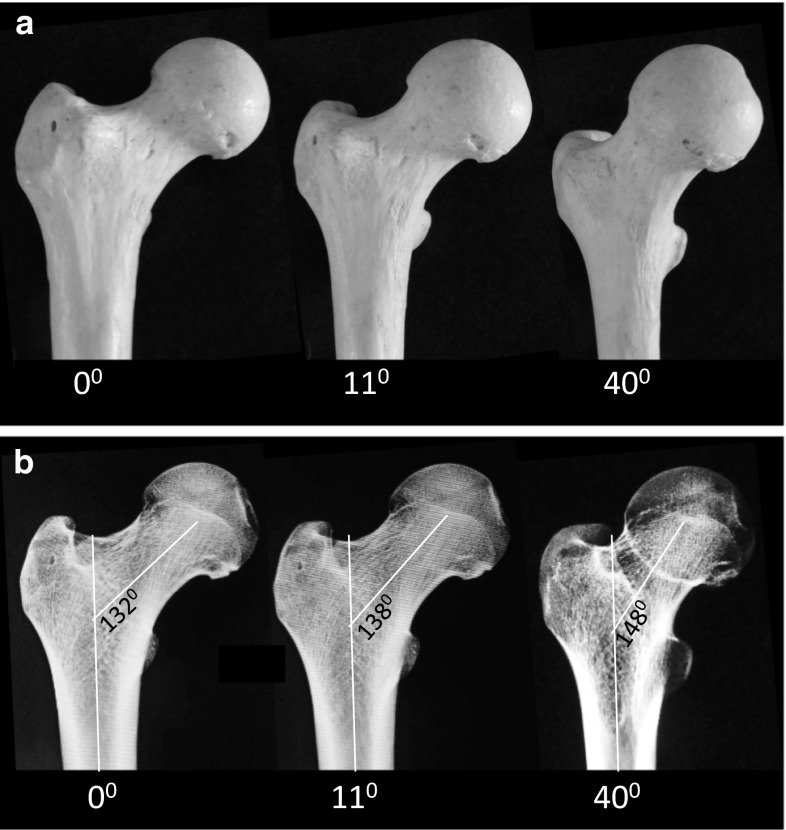
**a** As a femur is rotated from orthogonal to 11° of (normal) version, and to simulated 30° of excessive anteversion, the shape of the head and neck, along with the apparent CH–TD appears to change. The projection artifact may lead to spurious conclusions regarding the femoral head–neck offset. **b** The projected femoral neck-shaft is altered as a result of positioning, causing parallax distortion. This is why it is not possible to accurately document the neck-shaft angle on plain AP images. It would also lead the unwary to conclude that, by correcting anteversion with an antegrade intramedullary rod, there is an iatrogenic change in the neck-shaft angle. Therefore, we chose not to measure the neck-shaft angle in this series

The radiographic evaluation included a standing AP view of the legs, with the patellae neutral, upon which we documented limb length discrepancy. This was performed preoperatively and repeated prior to rod removal. We measured the center of femoral head to trochanteric tip vertical offset distance (normally = 0, Fig. 1). Intentionally, we did not record the pre- and postoperative femoral neck-shaft angles because there is projection artifact introduced by patient positioning and parallax distortion, making such measurements unreliable (Fig. 2a, b).

The indications for surgery included tripping, activity related anterior knee (and sometimes hip) pain and patellar mal-tracking or instability. Rotational correction was typically in the 30° range, with a minimum of 20° and maximum of 40°, leaving approximately 10°–15° of normal version of the femur. Patients with bilateral involvement, weighing less than 50 kg, were corrected simultaneously. Sixteen children with outward tibial torsion (pan genu torsion) underwent ipsilateral, simultaneous correction osteotomies. In select cases, concomitant lateral retinacular release (9 knees) or patellar realignment (4 knees) was combined with osteotomy (or undertaken at the time of rod removal). Iatrogenic retroversion was meticulously avoided.

## Technique

**Fig. 3:**
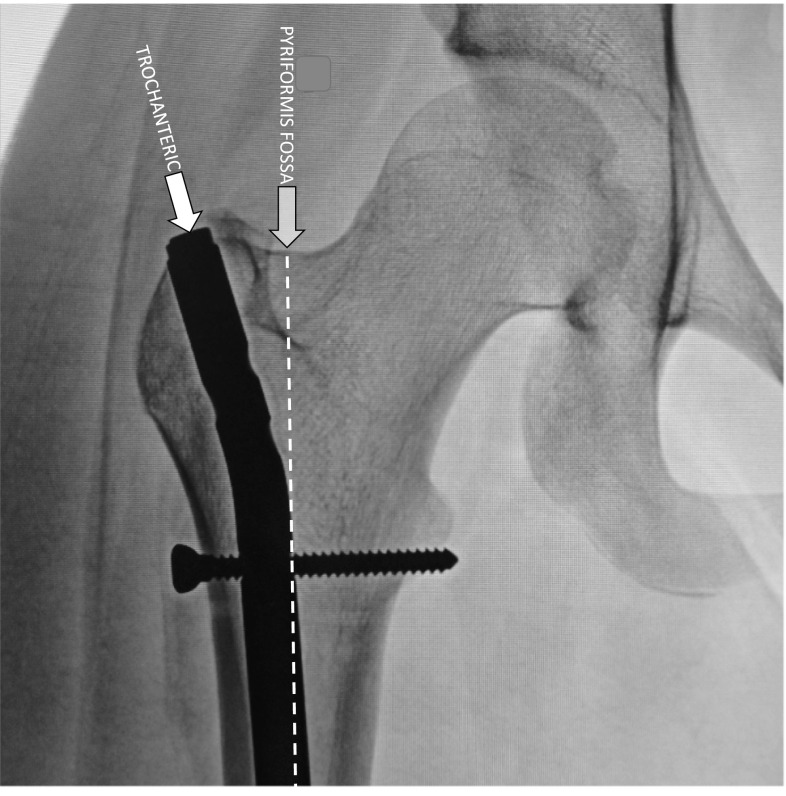
The new generation of trochanteric-entry, antegrade intramedullary femoral rods mitigates against iatrogenic osseous necrosis that had been previously reported with straight, piriformis entry rods. Presumably, the distance from the circumflex vessels protects against physical or thermal trauma during reaming

**Fig. 4:**
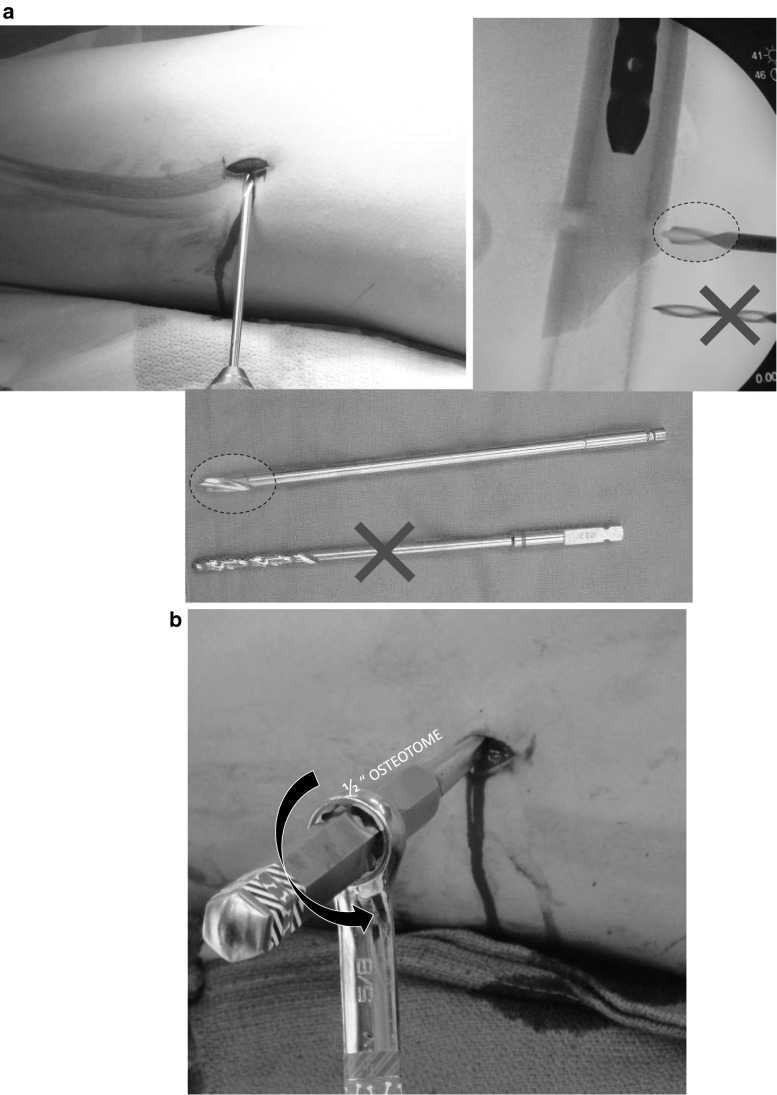
**a** Through a 1-cm incision, a stout drill bit (*dotted oval*), with a self-centering tip and short flutes, is employed to drill several transverse holes to mark the osteotomy site and decompress the femur for reaming. **b** These holes are connected with a ½-inch Ilizarov wrench (*hexagonal handle*) that is then torqued with a wrench. Advance the guide pin and rod past the osteotomy site *before* correcting the anteversion. Insert the distal interlocking screw first (freehand), then the proximal (jig)

The entry point for the nail is made, through a 3-cm incision, just lateral to the tip of the trochanter and well away from the piriformis fossa (Fig. 3). Prior to reaming, a 1-cm lateral mid-thigh incision is made and, using a stout 3.5- or 4.2-mm drill bit (Fig. 4a), several transverse drill holes are made for the dual purpose of decompressing the femoral canal and allowing egress of reaming material to serve as autogenous bone graft. The femur is then reamed sequentially until reaching a diameter that is 2 mm greater than the diameter of the rod to be used (typically 8 or 9 mm).

Rotation is monitored via two smooth 7/64th inch bi-cortical Steinman pins that are placed at the base of the greater trochanter (anterior or posterior to the rod) and one through the lateral femoral condyle. These are inserted at the angle of desired correction (using triangles to measure) relative to each other such that they will end up parallel once the osteotomy has been completed and the distal fragment rotated. The osteotomy is completed with a ½-inch osteotomy (hexagonal handle) that is torqued with a wrench to complete the cut (Fig. 4b). The distal interlocking screw is placed first under fluoroscopic guidance using the “free hand/perfect circle” technique. The proximal interlocking screw is introduced then using the jig.

Partial weight-bearing with crutches is encouraged; a wheelchair is preferred for school or long distance. With regional nerve block catheters delivering pain relief, patients are discharged the day after surgery typically. Crutch weaning commences 1 month (sooner with the newer locking bolt) following osteotomy and, if radiographs confirm callus formation, permission for progression of activities given.

## Results

### Clinical

The surgical goals were achieved primarily in all but two cases that required rod exchange for nonunion. In-toeing and tripping were relieved and anterior knee pain abated. Six patients required patellar realignment: four at the time of osteotomy and two subsequent to the rotational correction. There was no recurrence of torsion.

There were two femoral nonunions which required exchange of the rods. The remaining 55 patients had primary, solid radiographic union between 3 and 4 months.

In no instance was the center head to trochanteric distance altered by more than 5 mm, and limb lengths were not changed by more than 8 mm. Importantly, there were no cases of osseous necrosis of the femoral head or neck. This feared complication, reported in the literature, appears to be confined to patients with femoral fractures who are treated with an antegrade rod placed through the piriformis fossa. Some of the cases reported were diagnosed on follow-up radiographs without apparent clinical sequelae.

### Hardware issues

**Fig. 5:**
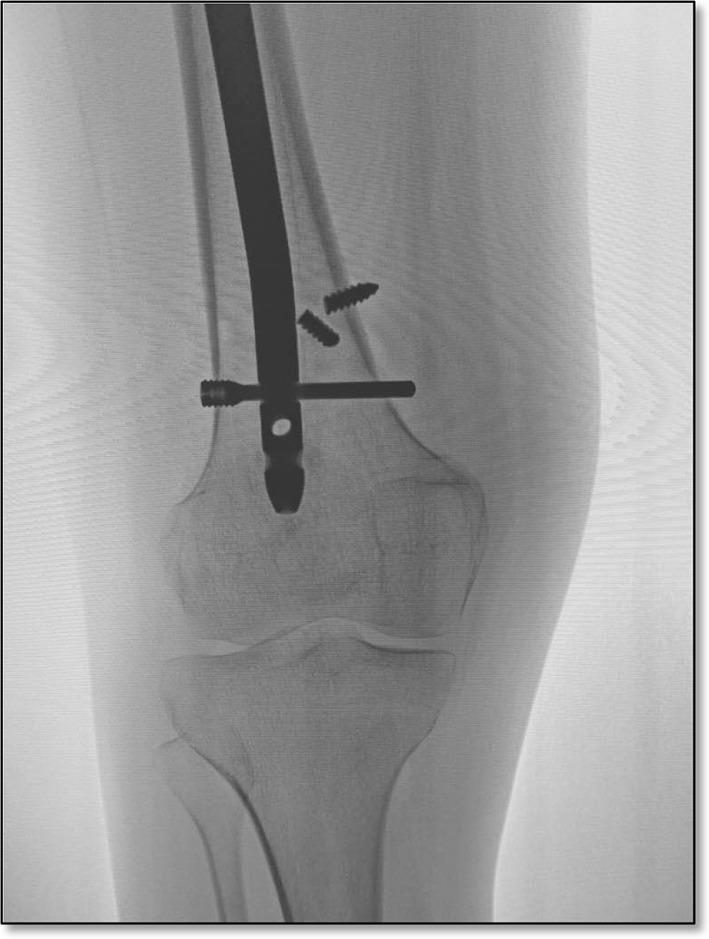
The core diameter of 3.0 mm for the screw has been increased to 3.7 mm for the bolt. None of the bolts have broken

There were 23 broken screws out of a total of 178 screws placed (including the two cases that underwent revision), giving a frequency of 12.5 %. This occurred typically between the second and fourth month postoperatively. The distal screw was more prone to failure by a ratio of 5:1. We surmise that, while both interlocking screws are subjected to the same vertical loading, the proximal screws are spared some of the rotational stresses due to the ball and socket nature of the hip. In contrast, the hinge action of the knee joint may impart more rotational stresses upon the distal screws. This problem appears to have been resolved through the use of stronger bolts (Fig. 5).

We were not able to correlate the patient’s BMI with the occurrence of broken screws. While early, protected weight-bearing was encouraged, we were unable to conclude that patient non-compliance or unauthorized activities were to blame for fatigue failure of the screws. None of the patients admitted to smoking tobacco. While this population may be at risk for dietary vitamin D deficiency, this was only documented on two patients (ages 13 and 15).

## Discussion

Femoral anteversion, which is normal in infants and younger children, should reduce spontaneously with growth. This does not merit bracing or physical therapy; parental education and periodic follow-up are appropriate in most cases. The natural history leads to spontaneous resolution of the problem by the age of 8 years in most. However, some patients have persistence of anteversion, manifested by in-toeing, tripping, anterior knee pain and even patellar instability. Persistent femoral anteversion (PFA) is more prevalent in females and may be familial. There remains a false perception by some practitioners that persistent anteversion is merely a cosmetic concern and that osteotomies are aggressive and unnecessary. However, with the emerging field of “jump mechanics” that is available in Movement Analysis Laboratories, evidence is coming to light that femoral anteversion is not innocuous [3–5]. Persistent anteversion may compromise hip kinematics and pose a rise of labral tears. At the knee, “dynamic valgus” puts increased stress on the patella and jeopardizes the integrity of the ACL. If unrecognized, the risks of failed knee surgery to correct these problems are increased [3].

In affected patients, a standard torsional profile (measured prone) will reveal both excessive inward femoral torsion (anteversion) and outward tibial torsion. If either (or both) reflects greater than 20° of malrotation, and when in a symptomatic child, corrective osteotomy may be warranted. This may be staged or performed simultaneously.

Radiographic evaluation should include a standing AP projection of the legs with the patellae facing forward. This will document the limb lengths and any deviation of the mechanical axis as well as revealing open physes. If the patello-femoral examination is abnormal or suspect, a sunrise view of the patella will reveal the sulcus along with lateral tilt and/or lateral subluxation of the patella. In specific patients, overt patellar mal-tracking or instability may warrant a lateral retinacular release or patellar tendon transfer at the time of the femoral rotational osteotomy. We no longer employ the “gunsight CT” scan due to concerns about radiation; gunsight MRI was briefly employed but subsequently was abandoned due to cost.

The optimal timing and staging of surgery must be individualized and is predicated upon thorough discussions with the parents regarding symptoms of tripping, anterior knee pain and, in some patients, patellar instability. While waiting until the age of 10 or older is preferable, some families prefer to intervene sooner. If there is pan genu torsion, due to concomitant tibial torsion, or overt patellar tilt or subluxation, these may be corrected simultaneously. Angular deviations (genu valgum or fixed knee flexion) may be addressed by simultaneous guided growth [6, 7].

The advantages of the percutaneous osteotomy are self-evident: the quadriceps and periosteum are spared from dissection; the osteotomy hematoma is contained within a closed space, expediting the formation of callus; the surgical scars are minimal, without the keloid formation seen sometimes with plates or blade plates [8].

With respect to the use of antegrade intramedullary rods in adolescent fractures (or osteotomies), the main concerns relate to the risk of iatrogenic osteonecrosis or disturbance of femoral neck growth [9]. Some authors have advocated against the use of antegrade rods in adolescents [10]. However, reported cases of osseous necrosis have been rare and subclinical, sometimes noted at the time of rod removal. Review of the literature implicates piriformis fossa entry techniques that were performed for introduction of straight rods [11, 12]. In an extensive meta-analysis of 1277 publications regarding locked antegrade rods in skeletally immature patients [13, 14], MacNeil et al. [15] extracted 19 relevant articles and concluded that the risk of necrosis was 2 % with piriformis entry, 1.4 % at the tip of the trochanter and 0 % utilizing a lateral trochanteric-entry site. This was the impetus for the development of the lateral trochanteric-entry point rods for trauma [1]. Stevens et al. [16] reported the use of the Philips intramedullary rod (Philips/Biomet) for 40 elective rotational osteotomies, with no observed growth disturbance in the femoral head or neck. The findings are consistent with recent trauma literature; in a series of 241 adolescent patients with 246 femoral fractures, Crosby et al. [17] reported only 2.2 % of clinically relevant growth disturbance and no femoral head osteonecrosis. We identified no clinically significant incidents of growth disturbance using the Pedi-Nail (Orthopediatrics, Warsaw, Indiana, USA) which is a lateral trochanteric-entry point device. We defined the threshold of “significant” as >5 mm change in the center of femoral head—tip of trochanter distance and >10 mm in relative femoral length as compared to the opposite side. Based upon our measurements of the center of femoral head to trochanteric tip distance, we concluded that the risk of causing proximal femoral growth disturbance in children and adolescent patients has been mitigated effectively by current implant designs that avoid the piriformis fossa perforation.

We describe the technique of percutaneous femoral rotational osteotomy of the femur, combined with an antegrade, trochanteric-entry intramedullary rod fixation. Despite the reported incidence of broken screws in this series, the outcomes were generally excellent with low morbidity. Since changing to a larger 3.7 mm unicortically threaded, interlocking bolts there have been no further incidences of fatigue failure. Problems experienced with extensile exposure and application of plate devices are averted, and osseous necrosis has not been observed with this technique.
